# Inflammatory biomarkers for predicting postoperative atrial fibrillation in cardiac surgery

**DOI:** 10.25122/jml-2025-0085

**Published:** 2025-05

**Authors:** Raluca-Elisabeta Staicu, Alina-Ramona Cozlac, Marius Emil Sintean, Alina Gabriela Negru, Florin Gorun, Sebastian Ciurescu, Corina Vernic, Ana Lascu, Petru Deutsch, Florin Horhat, Elena Cecilia Rosca

**Affiliations:** 1 Doctoral School Medicine-Pharmacy, Victor Babes University of Medicine and Pharmacy, Timisoara, Romania; 2 Clinic of Anesthesia and Intensive Care, Institute for Cardiovascular Diseases of Timisoara, Timisoara, Romania; 3 Department VI Cardiology–Cardiovascular Surgery, Victor Babes University of Medicine and Pharmacy, Timisoara, Romania; 4 Advanced Research Center, Institute for Cardiovascular Diseases, Victor Babes University of Medicine and Pharmacy, Timisoara, Romania; 5 Cardiology Department, Institute for Cardiovascular Diseases of Timisoara, Timisoara, Romania; 6 Obstetrics And Gynecology, Timisoara Clinical Municipal Emergency Hospital, Timisoara, Romania; 7 Department III Functional Sciences–Medical Informatics and Biostatistics, Victor Babes University of Medicine and Pharmacy, Timisoara, Romania; 8 Department III Functional Sciences–Pathophysiology, Victor Babes University of Medicine and Pharmacy, Timisoara, Romania; 9 Centre for Translational Research and Systems Medicine, Victor Babes University of Medicine and Pharmacy, Timisoara, Romania; 10 Cardiovascular Surgery Clinic, Institute for Cardiovascular Diseases of Timisoara, Timisoara, Romania; 11 Department of Surgery X, Victor Babes University of Medicine and Pharmacy, Timisoara, Romania; 12 Department of Microbiology, Victor Babes University of Medicine and Pharmacy, Timisoara, Romania; 13 Microbiology Department, Multidisciplinary Research Center on Antimicrobial Resistance (MULTIREZ), Victor Babes University of Medicine and Pharmacy, Timisoara, Romania; 14 Department of Neurology, Victor Babes University of Medicine and Pharmacy, Timisoara, Romania; 15 Department of Neurology, Clinical Emergency County Hospital, Timisoara, Romania

**Keywords:** inflammation, atrial fibrillation, cardiac surgery, IL-6, IL-17A, systemic inflammatory response index (SIRI), neutrophil-to-lymphocyte ratio (NLR), C-reactive protein

## Abstract

Postoperative atrial fibrillation (POAF) is a frequent complication of cardiac surgery associated with adverse outcomes. Systemic inflammation is implicated in POAF pathogenesis, suggesting inflammatory biomarkers may have predictive value. This study investigated the predictive capacity of readily accessible inflammatory markers for POAF during the early postoperative period in the cardiac intensive care unit, particularly within the 48–72-hour window when POAF most commonly occurs. In this prospective, single-center study, we enrolled 70 patients undergoing elective cardiac surgery with cardiopulmonary bypass. We measured preoperative and postoperative (24h, 48h) levels of neutrophil-to-lymphocyte ratio (NLR), systemic immune-inflammation index (SII), systemic inflammatory response index (SIRI), C-reactive protein (CRP), interleukin-6 (IL-6), and interleukin-17A (IL-17A). POAF was systematically monitored. We assessed the predictive value of these markers using ROC curve analysis and logistic regression, adjusting for clinical risk factors. The coronary cohort showed that the NLR at both 24 hours and 48 hours were the most discriminative markers for predicting POAF, with PCR at 48 hours achieving a moderate AUC of 0.66. In multivariate regression models, PCR at 48 hours (*P* = 0.009) and age (*P* = 0.046) emerged as significant predictors, while NLR and CPB duration were moderately correlated with the occurrence of POAF. In contrast, within the valvular patient subgroup, the NLR again exhibited promising predictive value, along with increased markers of tissue injury such as CK, LDH, and creatinine. Readily accessible postoperative inflammatory markers, particularly NLR at 24 hours and CRP at 48 hours, demonstrated moderate predictive value for POAF in patients undergoing elective cardiac surgery. These markers, especially NLR and CRP, may potentially contribute to improved POAF risk stratification in clinical practice when combined with clinical risk factors. Furthermore, our analysis also indicates that preoperative IL-17A levels may influence the occurrence of POAF. Therefore, alongside CRP and NLR, preoperative IL-17A can be considered a potentially significant marker for atrial fibrillation following cardiac surgery. However, these findings are preliminary and require validation in larger, multi-center studies to confirm their clinical utility and inform preventative strategies.

## INTRODUCTION

Open heart surgery, while life-saving, carries a significant risk of complications, ranging from hemorrhagic events (e.g., bleeding, cardiac tamponade), increased transfusion requirements, myocardial dysfunction/infarction, arrhythmias (e.g., branch block, atrioventricular block), pulmonary complications, acute kidney injury, gastrointestinal complications, neurological dysfunction, heart failure, wound infection, prolonged mechanical ventilation, and rare occurrences such as aortocoronary venous fistulas [1-3]. Among these, postoperative atrial fibrillation (POAF) stands out as a particularly concerning complication. POAF is a common and serious complication after cardiac surgery, affecting up to 35% of patients [4] and increasing morbidity, mortality, and healthcare costs. Despite advances in cardiac surgery, POAF remains a significant clinical challenge requiring better risk prediction and prevention strategies. The pathophysiology of POAF in this setting is complex, involving surgical trauma, cardiopulmonary bypass (CPB)-induced inflammation, ischemia-reperfusion injury, and atrial remodeling [5,6]. Systemic inflammation is now recognized as a central driver of POAF [7]. Therefore, identifying readily measurable biomarkers that reflect this inflammatory response holds promise for improving the prediction of POAF risk and targeted prevention. The primary objective of this study was to evaluate the role of specific inflammatory markers in predicting the onset of POAF in patients undergoing cardiac surgery. By investigating the potential association between these biomarkers and the development of POAF, we aimed to assess their predictive value and explore how they may contribute to risk stratification in this clinical setting. Secondary objectives of the study include:

- To assess the temporal dynamics of inflammatory markers before and after surgery, evaluating how the levels change over the perioperative period and how these changes correlate with the development of POAF.
- To investigate the relationship between inflammatory marker levels and other known risk factors for POAF, such as age, sex, and surgical approach, to understand better how these biomarkers may interact with traditional risk factors in predicting POAF.

By clearly articulating both the primary and secondary objectives, we aim to provide a comprehensive and systematic investigation of inflammatory markers in the context of POAF while also addressing potential confounders and further refining their clinical applicability. A range of inflammatory markers reflecting the body's response to surgical stress have been investigated for their potential to predict POAF. These include the neutrophil-to-lymphocyte ratio (NLR), C-reactive protein (CRP), interleukin-17A (IL-17A), and interleukin-6 (IL-6), all of which may promote atrial arrhythmias through mechanisms like myocardial inflammation, oxidative stress, and neurohormonal activation. Systemic immune-inflammation index (SII), systemic inflammatory response index (SIRI), NLR, and CRP are accessible systemic markers of inflammation and immune balance that may reflect the postoperative inflammatory state and predict POAF risk. Cytokines like IL-6 and IL-17A offer more specific insights into inflammatory pathways potentially relevant to POAF pathogenesis. Understanding the individual and comparative predictive utility of these markers requires considering their underlying mechanisms in the context of cardiac surgery-induced inflammation.

The neutrophil-to-lymphocyte ratio is known to increase during SIRS [8]. It is a systemic inflammatory marker derived from complete blood counts, reflecting the balance between neutrophils (pro-inflammatory innate immunity) and lymphocytes (regulatory and adaptive immunity). Elevated NLR may indicate systemic immune imbalance and increased pro-inflammatory activity relevant to surgical stress. In cardiac surgery, NLR elevation may reflect surgical stress, systemic inflammation, and immune dysregulation, all of which contribute to POAF. Postoperative surgical stress and cardiopulmonary bypass (CPB) can induce neutrophilia/lymphocytopenia. Elevated NLR may promote atrial remodeling and arrhythmogenesis [9]. Studies suggest that elevated NLR is associated with an increased risk of POAF [10], supporting its investigation as a potential predictive biomarker. While conditions common in elderly cardiac patients, such as diabetes [11] and hypertension [12], can influence NLR levels and potentially confound its interpretation [11], elevated NLR remains a significant serological indicator for assessing postoperative complication risk [8]. Emerging evidence suggests that preoperative NLR may serve as a valuable prognostic tool in cardiac surgery patients, although further research is needed to elucidate its role fully. Its significance in immune response is well-documented [13].

CRP is a classic acute-phase reactant, primarily synthesized by the liver in response to inflammatory cytokines, particularly IL-6 [14]. CRP is a widely recognized and clinically used biomarker of systemic inflammation, reflecting the overall burden of the acute phase response [15,16]. Its levels rapidly increase in response to tissue injury, infection, and inflammatory stimuli, making it a sensitive, albeit non-specific, indicator of systemic inflammatory activity. Research suggests a strong link between elevated CRP levels and POAF, although definitive large-scale studies are still needed. Elevated CRP levels around the time of surgery may contribute to atrial fibrillation (AF) development [17]. In the setting of cardiac surgery, CRP levels are predictably elevated in the postoperative period as part of the normal acute phase response to surgical trauma and CPB [18]. However, the magnitude and kinetics of CRP elevation may vary between individuals and in relation to POAF development. Elevated postoperative CRP levels have been consistently associated with an increased risk of POAF in numerous studies [19]. It is hypothesized that CRP, reflecting the systemic inflammatory burden, serves as a surrogate marker for the pro-inflammatory milieu that promotes atrial arrhythmogenesis after cardiac surgery. While CRP is not specific to atrial inflammation, its readily measurable nature and established association with POAF make it a clinically attractive biomarker candidate for risk prediction.

IL-17A is a pro-inflammatory cytokine primarily produced by T helper 17 (Th17) cells, a subset of CD4+ T lymphocytes that play a critical role in adaptive immunity and inflammatory responses [20]. IL-17A is implicated in a range of inflammatory and autoimmune diseases and is increasingly recognized for its involvement in cardiovascular inflammation and fibrosis [21]. IL-17A promotes the production of other pro-inflammatory cytokines, chemokines, and matrix metalloproteinases, including IL-6, TGF-β, and IL-1β [15,22], contributing to tissue inflammation and remodeling processes [23]. Preclinical studies in animal models indicate that blocking the IL-17A receptor can prevent POAF. Investigating the role of IL-17A in POAF may pave the way for novel anti-inflammatory therapies to reduce inflammation and potentially lower POAF incidence [24]. Given the central role of IL-17A in inflammation, preoperative IL-17A might reflect a pre-existing pro-inflammatory status relevant to POAF susceptibility, justifying its investigation.

IL-6, a pleiotropic cytokine, is a central mediator of inflammation and the acute phase response [25] and a key inducer of CRP [26]. Elevated IL-6 has been implicated in stroke associated with POAF and is involved in atrial remodeling, thrombus formation, and inflammatory processes [27]. Studies also demonstrate a direct correlation between higher IL-6 and increased complication rates in cardiac surgery patients, especially those with high EuroSCORE in intensive care settings [28]. Its role in atrial inflammation suggests potential predictive value [29,30], but we aim to clarify its independent contribution compared to readily available markers, such as CRP/NLR, in the context of POAF prediction.

The correlation between SII, SIRI, and various perioperative pathological conditions, including stroke and coronary artery disease [31], postoperative renal injury [32], the risk of diabetic cardiovascular complications [31], postoperative delirium [33] and POAF [34], has led to increasing recognition of these markers in clinical research and practice. The SII comprises three immune components: platelets, neutrophils, and lymphocytes, providing a more comprehensive picture of the thrombo-inflammatory status. In addition to inflammation, platelet activation and thrombogenesis also contribute to the pathophysiology of POAF, making SII a comprehensive marker of this condition. SIRI adds monocytes to the equation, highlighting the monocyte/macrophage inflammatory axis, which is important in chronic and post-operative inflammatory responses. Monocytes contribute to myocardial fibrosis and structural remodeling, both of which predispose to AF after surgery.

Knowledge gaps persist despite prior research, including inconsistent findings for individual markers, limited data on marker combinations, and the need for practical biomarkers. The optimal timing of marker measurement also remains uncertain. This research seeks to contribute evidence towards improving clinically useful POAF risk stratification.

This prospective study aimed to evaluate the predictive potential of a focused panel of inflammatory markers —NLR, CRP, IL-6, and IL-17A — for POAF in patients undergoing elective cardiac surgery with cardiopulmonary bypass during the first five postoperative days, taking into account the dynamics of these inflammatory markers after surgery [35,36]. The five-day time frame for studying these inflammatory markers was chosen to capture the peak postoperative inflammatory response most relevant to complications such as POAF while minimizing confounding from unrelated late postoperative events and improving the clinical utility of early risk stratification. After cardiac surgery, especially cardiopulmonary bypass, the inflammatory response peaks within the first 48-72 hours, and markers such as CRP, IL-6, NLR, SII, and SIRI typically rise sharply immediately postoperatively and begin to decline after 4-5 days. Beyond day 5, patients may develop new events (e.g., infection, wound healing response, secondary organ dysfunction) that may confound inflammatory marker levels. Limiting the analysis to the early postoperative period ensures that the measured inflammation is attributable to the surgery itself and not to unrelated complications. Currently, no reliable serological marker exists to predict the systemic inflammatory response syndrome (SIRS) after surgery [37] and its associated complications, including POAF. Our primary research objective was to determine whether preoperative and early postoperative levels of these inflammatory markers, assessed individually and in combination, could effectively identify patients at an elevated risk of developing POAF.

Our theoretical framework for inflammatory marker selection proposes that cardiac surgery and CPB trigger a systemic inflammatory response. We hypothesized that this inflammatory response mediates POAF mechanisms, ultimately leading to postoperative atrial fibrillation. The markers in our panel represent key aspects of this process, allowing for a multi-faceted assessment: SII, SIRI, CRP as an acute-phase marker, NLR as an indicator of immune balance, IL-6 as an early pro-inflammatory cytokine, and preoperative IL-17A potentially reflecting baseline pro-inflammatory status. We aimed to evaluate the individual and combined predictive utility of these factors for POAF. We selected this panel of markers (NLR, SII, SIRI, CRP, IL-6, IL-17A) because they cover both acute and chronic inflammation, reflect different immune cell lineages involved in the development of POAF, are easily calculated from standard blood tests, and based on their increased documented involvement in systemic inflammation and cardiac surgery outcomes. NLR, SII, and SIRI are composite indices derived from blood counts reflecting systemic immune balance [8,31,34]. CRP, an acute-phase reactant, and IL-6, a pro-inflammatory cytokine, are well-established predictors of cardiovascular complications post-surgery [14,27,28]. IL-17A, increasingly recognized for its role in atrial remodeling and fibrosis [20,22,24], provides insight into adaptive immune responses that may be implicated in POAF.

## MATERIAL AND METHODS

### Study design and patient population

This prospective, observational, single-center study was conducted at the Institute of Cardiovascular Diseases of Timisoara, within the Department of Cardiovascular Anesthesia and Intensive Care Medicine. Patient recruitment and data collection occurred between June 1, 2022, and November 1, 2023.

Inclusion criteria were adult patients (≥ 18 years of age) undergoing elective cardiac surgery requiring cardiopulmonary bypass, including coronary artery bypass grafting (CABG) and valve surgery. Exclusion criteria were the presence of preoperative atrial fibrillation documented in medical history or pre-operative electrocardiogram, preoperatively elevated inflammatory markers, history of chronic inflammatory diseases (e.g., rheumatoid arthritis, inflammatory bowel disease), autoimmune diseases (e.g., systemic lupus erythematosus), neoplastic diseases (active or within the past 5 years), severe renal dysfunction (eGFR < 30 mL/min/1.73m2), active infection at the time of surgery, American Society of Anesthesiologists (ASA) physical status more than III, preoperative hematocrit < 20%, and participation in other interventional clinical trials that could confound the study outcomes. Intraoperative exclusion criteria included hematocrit < 20%, hypothermia < 32°C, and intraoperative events such as mean arterial pressure (MAP) variations > 20% from baseline, MAP < 50 mmHg during CPB. Postoperative exclusion criteria included reinterventions and patients with postoperative infections or positive cultures. The presence of arrhythmias was defined as prior documentation of supraventricular tachycardia (SVT), premature atrial complexes (PACs), or premature ventricular complexes (PVCs), excluding preoperative atrial fibrillation, which was an exclusion criterion. These exclusion criteria were implemented to focus on the acute inflammatory response to cardiac surgery in a relatively homogeneous population, minimizing the influence of pre-existing inflammatory conditions on biomarker levels. However, this selection may limit the generalizability of our findings to higher-risk populations with pre-existing inflammation or a history of AF, a limitation we address in the discussion section.

For descriptive purposes and secondary subgroup analyses, patients were categorized post-hoc into two groups based on the primary surgical procedure performed: CABG and valve surgery. While this categorization was performed after data collection for presentation purposes, the distinction between CABG and valve surgery is a clinically relevant factor known to influence POAF risk and potential inflammatory responses.

Primary analyses were conducted on the entire cohort, with surgical type included as a covariate in multivariable models to adjust for potential differences in outcomes. Limitations of subgroup analyses due to unequal group sizes, particularly in the CABG subgroup, are acknowledged in the discussion. The smaller size of the CABG subgroup may affect the robustness of subgroup-specific findings, increasing the risk of type II error or overfitting in multivariate models. These limitations are acknowledged when interpreting subgroup-specific results.

### Surgical and anesthetic procedures

All patients underwent open-heart surgery involving valve surgery or CABG utilizing cardiopulmonary bypass. Myocardial protection was achieved using St. Thomas cardioplegia, which was administered at a blood/crystalloid ratio of 1:1 for priming and 4:1 for maintenance doses and delivered at 15-20-minute intervals. The priming solution included 500 mL of gelofusine, 500 mL of Ringer's, 20 milligrams of furosemide, 100 mg of heparin, 1000 milligrams of cefazolin, and 100 mL of 20% osmofundin. Our institution's cardioplegia circuit consists of a Sorin roller pump with 1- to 4-inch cardioplegia delivery tubing and a CSC 14 heat exchanger. Anesthesia was standardized across all patients, utilizing sevoflurane, propofol, sufentanil, midazolam, and rocuronium for general anesthesia, with adjunct medications including nitroglycerin, tranexamic acid and norepinephrine as clinically indicated.

### Postoperative atrial fibrillation monitoring and definition

POAF was systematically monitored for all patients from the time of surgery until discharge or for a maximum of 7 days postoperatively, whichever occurred first. Continuous electrocardiographic monitoring was performed in the intensive care unit (ICU) for at least 48 hours post-surgery, followed by at least twice-daily 12-lead electrocardiograms and continuous telemetry monitoring on the cardiac surgery ward. POAF was defined as any episode of atrial fibrillation or atrial flutter lasting ≥ 30 seconds, as documented by electrocardiogram, telemetry, or rhythm monitoring devices [38], adjudicated by a cardiologist blinded to biomarker data. The timing of POAF onset was recorded based on the first documented episode. The personnel analyzing inflammatory biomarker levels were blinded to patient outcomes, and data collectors recording postoperative atrial fibrillation were blinded to inflammatory biomarker results.

### Inflammatory marker assessment

Peripheral venous blood samples were collected at three pre-defined time points: preoperatively (within 24 hours prior to surgery, after admission and pre-anesthesia), 24 hours post-surgery (± 2 hours), and 48 hours post-surgery (± 2 hours). Additional samples were taken on postoperative days 3, 5, and 7, or at other clinically relevant time points, to monitor the trajectory of the inflammatory response and its association with the development of atrial fibrillation. For IL-6 and IL-17A measurements, additional venous blood samples were collected preoperatively and at 48 hours postoperatively. Samples were drawn into ethylenediaminetetraacetic acid (EDTA)-treated tubes, centrifuged immediately to separate plasma, and stored at –80 °C until analysis [39-41]. NLR, SII, and SIRI values were derived from complete blood counts, while CRP, IL-6, and IL-17A concentrations were measured directly. IL-6 and IL-17A concentrations were determined using commercially available Legend Max Human ELISA kits (BioLegend, Catalog Number: 430504). Reference ranges were defined as follows: NLR = 1.0–3.0, CRP < 3.0 mg/L, SII < 1000, SIRI = 1.0–1.5, IL-6 < 5.0 pg/mL, and IL-17A < 10–20 pg/mL.

The following formulas were applied: NLR = Neutrophil count / Lymphocyte count; SII = (Platelet count × Neutrophil count) / Lymphocyte count; SIRI = (Neutrophil count × Monocyte count) / Lymphocyte count. Delta values (ΔCRP, ΔNLR, ΔIL-6, ΔIL-17A) were defined as the difference between preoperative baseline values and 48-hour postoperative values.

All blood samples were collected between 06:00 and 08:00 AM to minimize circadian variability. Sample processing, including centrifugation and storage, was performed according to standardized operating procedures (SOPs) in the clinical laboratory.

For this study, only the preoperative, 24-hour, and 48-hour time points were analyzed. Samples collected on postoperative Days 3, 5, and 7 were stored for future exploratory analyses and were not included in the present study results.

### Data analysis

Statistical analyses were performed using SPSS version 27.0. Continuous variables are presented as mean ± standard deviation (SD) or median with interquartile range (IQR), depending on data distribution as assessed by the Shapiro–Wilk test. Categorical variables were presented as frequencies and percentages. Group comparisons were performed using *t*-tests or Mann–Whitney U tests for continuous variables and chi-square tests or Fisher's exact test for categorical variables, as appropriate. To control for Type I errors in multiple comparisons and correlation analyses, the Holm–Bonferroni correction was applied. To evaluate the predictive capacity of inflammatory markers for postoperative atrial fibrillation, we employed the following statistical approaches:

#### Receiver operating characteristic (ROC) curve analysis

Receiver operating characteristic (ROC) curve analysis was conducted for each marker (SII, SIRI, NLR, CRP, IL-6, and IL-17A) at the preoperative, 24-hour, and 48-hour time points to assess its ability to discriminate between patients who developed POAF and those who did not. The area under the curve (AUC) was calculated with 95% confidence intervals and interpreted according to established thresholds: 0.5–0.7 indicating poor, 0.7–0.8 acceptable, 0.8–0.9 excellent, and values above 0.9 considered outstanding discrimination. Optimal cut-off values for each biomarker were determined using Youden’s index during ROC curve analysis; however, the primary analyses focused on continuous marker values.

#### Logistic regression analysis

Univariate and multivariable logistic regression analyses were conducted to determine the association between inflammatory markers and POAF, adjusting for potential confounders. In the univariate analysis, each inflammatory marker (at all time points) and established clinical risk factors (age, diabetes mellitus, surgical procedure type, CPB time, blood transfusion) were initially assessed in separate univariate logistic regression models. A multivariable logistic regression model was then constructed with a priori covariates: age, diabetes mellitus, surgical procedure type, CPB time, and blood transfusion. Postoperative CRP at 48 hours, NLR at 24 hours, and preoperative IL-17A (selected based on univariate findings, clinical accessibility, and hypotheses) were then individually added to this base model to assess their independent predictive value. Adjusted odds ratios (ORs) with 95% confidence intervals and *P* values are reported. Events Per Variable (EPV) ratios were monitored to assess model stability. Multivariable logistic regression models included all pre-specified covariates (age, diabetes, surgical type, CPB time, blood transfusion) irrespective of their statistical significance in univariate analysis to account for potential confounding. We adopted a threshold of ≥10 events per variable (EPV) for model stability, and this threshold was met for the primary multivariable logistic regression models.

#### Analysis focusing on pre-POAF onset markers

Secondary analyses focused on inflammatory markers measured preoperatively and 24 hours postoperatively to assess predictive value before typical POAF onset (within 48–72 hours).

#### Sensitivity analyses stratified by blood transfusion status

Sensitivity analyses were performed to examine whether the associations between key inflammatory markers and POAF were modified by blood transfusion status (transfused vs. non-transfused) by stratifying ROC and regression analyses by transfusion status. Due to sample size limitations, transfusion status was explored using stratified analyses rather than formal interaction testing in multivariable models. Future studies with larger cohorts should explore effect modification through interaction terms.

#### Post-hoc power analysis

A post-hoc power analysis was conducted based on the observed effect sizes. For detecting moderate correlations (r = 0.33) between inflammatory markers and POAF with α = 0.05, the sample size of 70 patients achieved approximately 80% statistical power. We acknowledge that post-hoc power analyses, although informative, have inherent limitations, particularly when derived from observed non-significant findings, and should be interpreted with caution.

Statistical significance was defined as a *P* value < 0.05 after Holm-Bonferroni correction where applicable unless otherwise specified. Confidence intervals of 95% are reported for correlation coefficients, AUC values, and odds ratios to indicate the precision of estimates. Patients with missing critical data, such as incomplete ECG recordings for POAF monitoring or absent 24-hour/48-hour inflammatory marker measurements, were excluded from the final statistical analysis.

## RESULTS

### Patient characteristics

Baseline characteristics of the study population are summarized in Tables 1 and 2. A total of 70 patients undergoing elective cardiac surgery with cardiopulmonary bypass were enrolled. The mean age was 68.3 ± 9.5 years, and 28.6% were women. Diabetes mellitus was present in 31.4% of the cohort. CABG was the primary surgical procedure in 32.9% (*n* = 23) of patients, and valve surgery in 67.1% (*n* = 47), with a mean CPB time of 117.46 minutes (SD = 30.26).

**Table 1:** Baseline characteristics of the study population

Variable	Total (*n* = 70)
Gender (Female), *n* (%)	15 (21.42%)
Age	62.06 + 12.06
Age group, *n* (%)	
≤ 55 years	13 (18.57%)
55 to 65 years	20 (28.57%)
65 to 75 years	32 (45.71%)
≥ 75 years	5 (7.14%)
Height, cm	170.90 ± 9.93
Weight, kg	80.83 ± 16.4
BMI, kg/m^2^	27.55 ± 4.54
BMI group, *n* (%)	
≤ 18.5 kg/m^2^	0 (0%)
18.5 to 24 kg/m^2^	17 (24.28%)
≥ 24 kg/m^2^	53 (75.72%)
Coronary heart disease, *n* (%)	23 (32.85%)
Diabetes mellitus, *n* (%)	20 (28.57%)
Arrhythmias, *n* (%)	27 (38.57%)
Surgical approaches, *n* (%)	
Valve surgery	47 (67.14%)
Coronary surgery	23 (32.86%)
CPB time, min	117.46 ± 30.26

BMI, Body Mass Index; CPB, Cardiopulmonary Bypass Time.

**Table 2:** Laboratory results in patients included in the study

Parameters	Min	Max	Mean value	Standard deviation
MV/hours	8	32.00	16.84	5.35
SII	311	7711	2359.54	486
SIRI	2.5	51.5	13.42	486.17
CRP at 24h	40.3	135.00	75.47	21.35
CRP at 48h	69.2	376.00	190.13	68.64
ΔCRP	13	273.00	114.67	55.61
NLR pre-operator	0.9	8.40	2.75	1.37
NLR at 24h	5.4	46.00	16.65	9.24
NLR at 48h	3.83	85.00	10.56	10.12
ΔNLR at 24h	3.3	42.90	13.90	9.14
ΔNLR at 48h	0.33	80.98	7.81	10.00
IL6 pre-operator	0	46.00	15.65	11.89
IL17-A pre-operator	0	234.20	5.08	28.12
IL6 at 48h	19.96	490.40	171.52	116.35
ΔIL-6	19.46	453.40	155.88	107.08
IL17-A at 48h	0	377.00	12.53	46.48
ΔIL17-A	0	142.80	7.45	19.75
MAP	75	120.00	90.54	9.76
CVP	6	14.00	9.31	1.95
B.T.	0	1.00	0.63	0.49
NA/hours	0	48.00	20.44	10.24
EF %	20	55.00	45.97	6.44
Blood Glucose at 24h	89	260.00	155.11	37.24

BMI, Body Mass Index; CABG, Coronary Artery Bypass Graft Surgery; CPB, Cardiopulmonary Bypass Time; MV, Mechanical Ventilation; MAP, Mean Arterial Pressure; CVP, Central Venous Pressure; BT, Blood Transfusion; NA, Noradrenaline; EF, Ejection Fraction.

Group 1 included 23 patients who underwent CABG, while Group 2 comprised 47 patients who underwent aortic or mitral valve replacement for stenosis and/or regurgitation.

The mean value of CRP at 48h was 190.13 mg/L with a standard deviation of 68.64, while IL-6 at 48h averaged 171.52 pg/mL (SD = 116.35). NLR at 24h had a mean of 16.65 (SD = 9.24), and Delta IL-6 showed a mean increase of 155.88 pg/mL (SD = 107.08). The mean SII value was 2359.54, and the SIRI averaged 13.42 (SD = 486.17, Table 2).

### Postoperative atrial fibrillation incidence

The overall incidence of POAF in the study cohort was 38.6% (*n* = 27). When stratified by surgical procedure, the incidence of POAF was 34.8% (*n* = 8) in the CABG group (Group 1) and 40.4% (*n* = 19) in the valve surgery group (Group 2). The median time to POAF onset was 48 hours (interquartile range: 36–60 hours) postoperatively.

### Inflammatory marker response post-cardiac surgery

The time course of inflammatory markers (CRP, NLR, SII, SIRI, IL-6, and IL-17A) following cardiac surgery was assessed across both groups. Patients in Group 1 had a mean CPB duration of 104.47 ± 30.75 minutes. CRP levels increased from a mean of 78.67 ± 20.06 mg/L at 24 hours to 190.71 ± 71.10 mg/L at 48 hours. NLR values were 15.05 ± 9.02 at 24 hours and decreased to 8.29 ± 5.07 at 48 hours. SII showed an increase from mean preoperative values of 462.47 to 2487.62 postoperatively. Regarding SIRI, we observed increases from mean preoperative values of 1.27 to mean postoperative values of 13.25. IL-6 levels increased from 15.76 pg/ml preoperatively to 166.29 pg/ml at 48 hours, while IL-17A increased from 0.81 pg/ml to 5.16 pg/ml. Eight patients (34.7%) in Group 1 developed POAF, with most cases occurring within 48 hours postoperatively (Table 3).

**Table 3:** Characteristics and laboratory tests of patients in Group 1

Variable	Total	Atrial fibrillation Yes	Atrial fibrillation No	*P* value
Diabetes - Yes	8 (25.0%)	3 (33.3%)	5 (21.7%)	0.8204
Smoking - Yes	10 (31.2%)	3 (33.3%)	7 (30.4%)	1.0
Age	62.53 (9.25)	62.11 (12.70)	62.70 (7.85)	0.9
Weight	86.84 (15.13)	84.78 (17.60)	87.65 (14.41)	0.6702
Height	174.31 (8.66)	171.00 (11.16)	175.61 (7.37)	0.2767
IMC	28.19 (3.86)	28.83 (3.74)	27.94 (3.97)	0.5632
CPB duration	104.47 (30.75)	127.56 (38.60)	95.43 (22.07)	0.0403
MV hours	15.78 (5.26)	19.11 (6.99)	14.48 (3.86)	0.0897
PCR at 24h	78.67 (20.06)	95.13 (11.76)	72.23 (19.04)	0.0004
PCR at 48h	190.71 (71.10)	266.19 (48.57)	161.17 (54.98)	0.0001
ΔPCR	112.04 (59.06)	171.06 (43.40)	88.95 (47.42)	0.0002
NLR presurgery	2.42 (1.11)	2.47 (0.59)	2.39 (1.26)	0.8084
NLR at 24h	15.05 (9.02)	19.77 (10.86)	13.20 (7.69)	0.1249
NLR at 48h	8.29 (5.07)	10.61 (7.59)	7.38 (3.49)	0.2501
ΔNLR 24h	12.63 (8.93)	17.29 (10.62)	10.81 (7.68)	0.1224
ΔNLR 48h	5.87 (4.89)	8.13 (7.33)	4.99 (3.36)	0.2459
IL-6 presurgery	15.76 (12.36)	13.66 (9.82)	16.58 (13.33)	0.5037
IL17 presurgery	0.81 (1.66)	1.82 (2.67)	0.42 (0.85)	0.1591
IL-6 at 48h	166.29 (116.85)	145.45 (91.37)	174.45 (126.32)	0.4796
ΔIL-6	150.54 (106.25)	131.80 (83.63)	157.87 (114.73)	0.486
IL-17 at 48h	5.16 (8.53)	7.33 (9.38)	4.31 (8.24)	0.4124
ΔIL-17	4.34 (7.43)	5.51 (6.98)	3.88 (7.71)	0.574
MAP	89.84 (8.75)	92.78 (11.76)	88.70 (7.26)	0.3531
Creatinine pre-surgery	1.03 (0.44)	1.26 (0.72)	0.93 (0.24)	0.2174
Creatinine post-surgery	1.24 (0.54)	1.54 (0.91)	1.12 (0.25)	0.2049
WBC	11.54 (3.98)	14.95 (3.85)	10.21 (3.21)	0.0062
CK	628.22 (499.54)	875.11 (531.89)	531.61 (462.65)	0.1125
CK-MB	32.00 (28.16)	49.56 (47.88)	25.13 (10.43)	0.1665
LDH	303.72 (102.22)	386.22 (123.21)	271.43 (73.00)	0.025
CVP	8.59 (1.98)	10.00 (2.45)	8.04 (1.49)	0.0481
EF	47.28 (6.68)	42.00 (10.71)	49.35 (2.29)	0.0745
Blood glucose at 24h	145.94 (34.01)	166.56 (42.35)	137.87 (27.13)	0.0867
SII pre-surgery	462.47 (161.31)	463.67 (191.47)	462.00 (152.76)	0.9817
SII post-surgery	2487.62 (1513.02)	2053.11 (639.52)	2657.65 (1723.24)	0.1583
SIRI pre-surgery	1.27 (0.98)	1.04 (0.59)	1.36 (1.10)	0.3028
SIRI post-surgery	13.25 (7.75)	12.52 (6.05)	13.53 (8.43)	0.7108

CPB, Cardiopulmonary Bypass Time; MV, Mechanical Ventilation; MAP, Mean Arterial Pressure; CVP, Central Venous Pressure; EF, Ejection Fraction; WBC, White Blood Cell count; CK, Creatine Kinase; LDH, Lactate Dehydrogenase.

Among the variables analyzed, creatine kinase-MB (CK-MB) demonstrated a statistically significant difference between groups, with higher values observed in patients who developed atrial fibrillation at 48 hours (*P* < 0.05). Additionally, CRP at 48h and ΔCRP showed significantly elevated mean levels in the atrial fibrillation group compared to those without, also reaching statistical significance (*P* < 0.05; Table 3).

**Figure 1:**
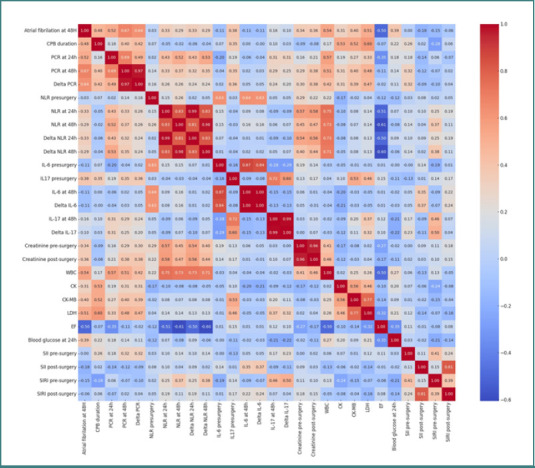
Correlation matrix heatmap illustrating the relationships between postoperative inflammatory markers and atrial fibrillation at 48 hours in Group 1

The correlation coefficients were as follows: r = 0.67, *P* < 0.05, for the association between POAF and CRP levels at 48 hours, and r = 0.55, *P* < 0.05, for POAF and CRP levels at 24 hours postoperatively. NLR demonstrated a correlation coefficient with POAF of 0.33 at 24 hours and 0.29 at 48 hours. Regarding the correlation between POAF and lactate dehydrogenase (LDH), we obtained r = 0.5, *P* < 0.05. The preoperative creatinine value was associated with r = 0.34, *P* < 0.05. The relationship between IL-17A and POAF yielded r = 0.38, *P* < 0.05 (Figure 1).

Statistical analysis in this patient cohort revealed a significant correlation between CRP and IL-17A and ejection fraction, with correlation coefficients of r = 0.7 (*P* ≤ 0.05) for CRP and r = 0.63 (*P* ≤ 0.05) for IL-17A. Patients who developed POAF had a mean EF of 40.37%, indicating that lower EF values were associated with a higher risk of POAF and an enhanced inflammatory response. In Group 2 (valve surgery patients), the mean CPB time was 120.95 ± 34.83 minutes. CRP levels were 73.38 mg/L at 24 hours and 184.27 mg/L at 48 hours. NLR values were 17.93 at 24 hours and 11.44 at 48 hours. IL-6 increased from 15.83 pg/ml preoperatively to 168.79 pg/ml at 48 hours, while IL-17A increased from 5.96 pg/ml to 13.33 pg/ml at 48 hours. SII showed an increase from 466.98 to 2235.16 48 hours postoperatively, and SIRI increased from 1.4 to 13.75 in the postoperative period (Table 4).

**Table 4:** Characteristics and laboratory tests of patients in Group 2

Variable	Total	Atrial fibrillation (Yes)	Atrial fibrillation (No)	*P* value
Diabetes - Yes	15 (26.8%)	10 (45.5%)	5 (14.7%)	0.0258
Age	61.73 (12.87)	66.45 (8.97)	58.68 (14.15)	0.0148
Weight	78.21 (15.29)	80.36 (15.32)	76.82 (15.34)	0.4031
Height	169.20 (9.08)	170.59 (8.70)	168.29 (9.33)	0.3532
IMC	27.20 (4.78)	27.64 (4.43)	26.91 (5.04)	0.5745
CPB duration	120.95 (34.83)	130.95 (38.90)	114.47 (30.81)	0.102
MV hours	19.04 (20.93)	23.73 (32.63)	16.00 (5.33)	0.2829
PCR at 24h	73.38 (22.77)	74.56 (27.71)	72.61 (19.34)	0.7753
PCR at 48h	184.27 (62.80)	192.51 (78.97)	178.93 (50.29)	0.4781
ΔPCR	110.89 (50.30)	117.95 (61.29)	106.32 (42.10)	0.4415
NLR presurgery	2.89 (1.39)	3.24 (1.78)	2.66 (1.02)	0.1708
NLR at 24h	17.93 (11.48)	21.90 (14.58)	15.36 (8.18)	0.0649
NLR at 48h	11.44 (11.52)	15.29 (17.43)	8.94 (3.41)	0.1057
ΔNLR 24h	15.04 (11.32)	18.65 (14.38)	12.70 (8.24)	0.088
ΔNLR 48h	8.55 (11.40)	12.04 (17.28)	6.29 (3.62)	0.1374
IL-6 presurgery	15.83 (10.62)	16.97 (9.97)	15.08 (11.10)	0.5113
IL17 presurgery	5.96 (31.42)	13.04 (49.76)	1.38 (3.83)	0.2852
IL-6 at 48h	168.79 (108.24)	178.00 (103.35)	162.83 (112.41)	0.6067
ΔIL-6	152.97 (100.55)	161.03 (95.41)	147.75 (104.81)	0.6268
IL-17 at 48h	13.23 (51.81)	25.64 (81.12)	5.20 (10.78)	0.2527
ΔIL-17	7.27 (21.67)	12.61 (32.94)	3.82 (7.81)	0.2316
MAP	88.80 (9.67)	88.86 (10.79)	88.76 (9.03)	0.9717
Creatinine pre-surgery	1.19 (0.85)	1.36 (1.09)	1.07 (0.63)	0.2652
Creatinine post-surgery	1.44 (1.06)	1.71 (1.41)	1.26 (0.74)	0.1791
WBC	12.05 (3.91)	12.71 (3.70)	11.62 (4.04)	0.3024
CK	659.59 (640.62)	759.32 (854.91)	595.06 (456.02)	0.4144
CK-MB	42.89 (21.34)	45.64 (26.32)	41.12 (17.60)	0.4832
LDH	389.57 (117.46)	425.55 (96.02)	366.29 (125.33)	0.0511
PVC	9.12 (2.03)	9.59 (2.28)	8.82 (1.82)	0.192
EF	46.34 (5.52)	44.77 (5.23)	47.35 (5.54)	0.0846
Blood glucose at 24h	160.34 (34.50)	177.41 (36.14)	149.29 (28.86)	0.0039
SII pre-surgery	466.98 (291.48)	459.18 (307.35)	472.03 (285.34)	0.8759
SII post-surgery	2235.16 (1432.28)	2096.86 (1044.12)	2324.65 (1644.47)	0.5288
SIRI pre-surgery	1.44 (1.33)	1.20 (0.99)	1.60 (1.51)	0.2319
SIRI post-surgery	13.75 (9.50)	11.38 (5.96)	15.28 (11.03)	0.0934

**Figure 2:**
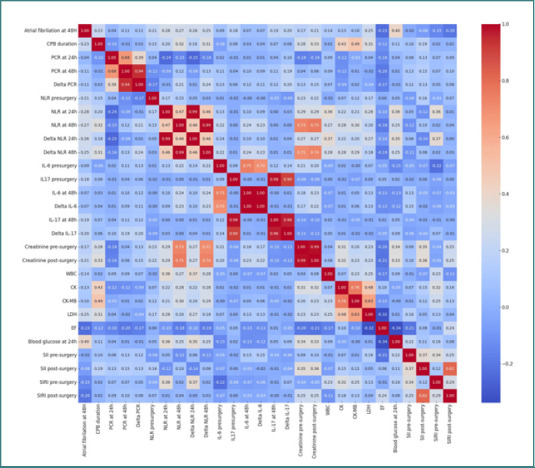
Correlation matrix heatmap illustrating the relationships between postoperative inflammatory markers and atrial fibrillation at 48 hours in Group 2

In Group 2 (valvular patients), those who developed POAF had a mean age of 66.45 years and a mean weight of 80.36 kg. Their mean CRP level at 48 hours was 192.51 mg/L, mean NLR was 15.29, and mean IL-6 at 48 hours was 178.00 pg/mL. In contrast, patients who did not develop POAF had a lower mean age of 58.68 years and a mean weight of 76.82 kg. Correlations between inflammatory markers and POAF in this group were generally weaker. CRP demonstrated a correlation with POAF of 0.04 at 24 hours and 0.11 at 48 hours, respectively. NLR correlations with POAF were 0.21 preoperatively and 0.28 at 24 hours (Figure 2).

**Figure 3:**
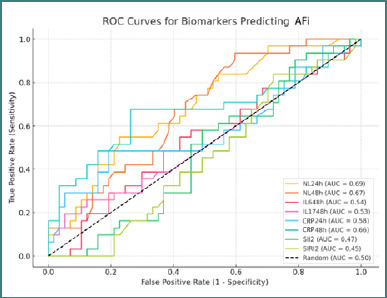
ROC curves for biomarkers predicting AFi in Group 1

Furthermore, in evaluating the predictive utility for postoperative complications, the coronary cohort (Group 1) demonstrated that NLR at both 24 hours (AUC = 0.69) and 48 hours (AUC = 0.67) were the most discriminative markers for predicting POAF. CRP at 48 hours also showed moderate predictive ability, with an AUC of 0.66 (Figure 3).

**Figure 4:**
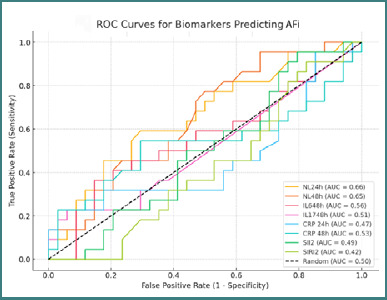
ROC curves for biomarkers predicting AFi in Group 2

For Group 2, NLR at 24 hours (AUC = 0.66) and 48 hours (AUC = 0.65) remained the most discriminative markers for POAF prediction (Figure 4).

In the multivariate regression models, PCR at 48 hours (*P* = 0.009) and age (*P* = 0.046) emerged as significant predictors, while NLR ratios (with correlation coefficients ranging from 0.28 to 0.31) and CPB duration (r ≈ 0.32) were moderately correlated with the occurrence of POAF. In contrast, within the valvular patient group (Group 2), the NLR ratios again exhibited promising predictive performance (with AUC values of approximately 0.66 at 24 hours and 0.65 at 48 hours), although PCR at 48 hours was notably weaker (AUC = 0.53). In this subgroup, age was the only significant predictor (*P* = 0.018), while the other inflammatory markers did not reach statistical significance. When comparing patients with and without POAF, those who presented the complication tended to be older, experienced longer CPB times, and required extended periods of mechanical ventilation and vasopressor support. Additionally, these patients had higher postoperative levels of inflammatory markers, such as CRP and NL ratios, as well as increased markers of tissue injury, including CK, LDH, and creatinine.

## DISCUSSION

This prospective study investigated the role of inflammatory markers in predicting POAF following cardiac surgery. Our findings suggest that while the overall inflammatory response is implicated in POAF, among the individual markers assessed, CRP at 48 hours and NLR at both 24 and 48 hours demonstrated the most consistent association with POAF occurrence in our cohort. Furthermore, our analysis also indicates that preoperative IL-17A levels may influence the occurrence of POAF. Therefore, alongside CRP and NLR, preoperative IL-17A can be considered a potentially significant marker for atrial fibrillation following cardiac surgery.

This elevation in NLR, a readily available and cost-effective marker, suggests a heightened postoperative inflammatory state or an altered immune balance that might predispose patients to POAF following surgical stress. This finding aligns with the growing body of evidence supporting NLR as a valuable biomarker for risk stratification in cardiovascular conditions, including atrial fibrillation. Indeed, in our coronary cohort (Group 1), NLR at both 24 hours (AUC = 0.69) and 48 hours (AUC = 0.67) were the most discriminative markers for predicting POAF. Postoperative CRP values, particularly at 48 hours, achieved a moderate AUC of 0.66 in the coronary cohort and were also found to be significant predictors of POAF, especially in the CABG group, where they emerged as a significant predictor in multivariate regression models (*P* = 0.009). We observed numerically higher postoperative CRP and NLR values at 24 and 48 hours in patients who developed POAF in both groups. Our study highlights the potential of NLR at 24 and 48 hours and CRP at 48 hours as informative markers in predicting the risk of POAF. We observed elevated postoperative NLR values in CABG and valve surgery groups who subsequently developed POAF. This elevation in NLR, a readily available and cost-effective marker, suggests a heightened postoperative inflammatory state or an altered immune balance that might predispose patients to POAF following surgical stress. Indeed, in our coronary cohort (Group 1), NLR at both 24 hours (AUC = 0.69) and 48 hours (AUC = 0.67) was the most discriminative marker for predicting POAF. While the study demonstrates an association between NLR and the incidence of POAF, the implications for clinical practice require further interpretation. Given that an elevated NLR is associated with an increased risk of POAF, its measurement could potentially serve as a useful prognostic tool to identify patients at higher risk. However, before NLR is incorporated into routine practice, it is important to establish standardized thresholds for what constitutes an 'elevated' NLR and to validate these thresholds in larger, more diverse patient populations. In terms of management, an elevated NLR may warrant closer monitoring of patients for signs of POAF, particularly in high-risk surgical populations. It may also guide early interventions aimed at reducing systemic inflammation, such as optimizing perioperative management strategies (e.g., controlling hyperglycemia, using anti-inflammatory medications, or adjusting the duration of cardiopulmonary bypass). However, NLR alone should not dictate management decisions, as it is likely a marker of broader inflammatory processes rather than a direct cause of POAF. Instead, its use should be considered in conjunction with other clinical variables (e.g., age, comorbidities, and other inflammatory markers) to inform a comprehensive risk stratification approach. Finally, further prospective studies are needed to determine whether routine measurement of NLR improves patient outcomes by allowing earlier intervention or tailored management strategies and to assess whether it has independent predictive value beyond other established risk factors for POAF.

Postoperative CRP values, particularly at 48 hours, achieved a moderate AUC of 0.66 in the coronary cohort and were also found to be significant predictors of POAF, especially in the CABG group, where they emerged as a significant predictor in multivariate regression models (*P* = 0.009). While other markers, such as IL-6 and postoperative IL-17A, showed postoperative increases, their predictive capacity for POAF in this study was less clear, highlighting the complex and multifactorial nature of POAF pathogenesis. Also, our analysis confirms that IL-6 and IL-17A demonstrated high variability, which limits their utility as standalone predictors in our cohort.

Consistent with existing literature, our study confirmed age as a significant predictor of POAF, with statistical significance observed in both the coronary cohort (*P* = 0.046) and the valvular cohort (*P* = 0.018). While prolonged CPB duration is frequently reported as a contributing risk factor for POAF, our findings support this association as well, with a moderate correlation between CPB time and POAF occurrence (r = 0.32, *P* < 0.05). In the CABG group, patients with POAF had a 25% longer CPB time (133.25 vs. 99.87 minutes), indicating that extended CPB may be a risk factor in this population. However, in the valvular surgery group, the differences in CPB duration were minimal, suggesting that other factors, such as age and underlying comorbidities, may influence the development of POAF more significantly in this subgroup. This observation emphasizes the multifactorial nature of POAF, where postoperative inflammatory responses, rather than CPB time alone, may play a pivotal role in our hemodynamically stable cohort. CPB duration is often interpreted as a proxy for the extent of surgical trauma, ischemic time, and inflammatory response, all of which can contribute to POAF pathogenesis. However, the duration should be contextualized in relation to other procedural variables, such as the type of surgery (e.g., coronary artery bypass grafting vs. valve replacement), patient comorbidities, and perioperative management. The relationship between CPB duration and POAF risk is typically non-linear, with prolonged CPB times being associated with an increased risk of POAF due to factors such as increased systemic inflammation, myocardial injury, and atrial structural remodeling. This aligns with the growing emphasis on inflammation as a central mechanism in the development of POAF [2-4].

Our study also supports the type of surgery as a significant factor influencing the risk of POAF. We observed a numerically higher POAF incidence in valve surgery patients (40.4%) compared to CABG patients (34.7%). This suggests that valve surgery, potentially due to its inherent complexities or specific inflammatory triggers compared to CABG, may confer a higher POAF risk. Our CRP findings, showing a postoperative increase as expected, highlight that certain limitations exist about relying on CRP alone to predict POAF across all cardiac surgery types. Despite a more pronounced CRP increase in the CABG group, we did not find significant differences in CRP levels between POAF and non-POAF patients in all subgroups, particularly in the valve surgery group, where its predictive value was weaker (AUC ≈ 0.53). While meta-analyses and several studies [42-44] establish CRP as an inflammatory marker associated with POAF and sometimes as an independent predictor, our data suggests that CRP, when assessed in isolation, may not fully capture the intricate inflammatory pathways driving POAF across different surgical contexts. The complex pathogenesis of POAF likely involves a broader inflammatory milieu than can be reflected by CRP alone. Although CRP is widely acknowledged as an inflammatory marker associated with POAF, our study noted surgery-specific predictive responses, with CRP performing better in the coronary.

While literature extensively links elevated IL-6 to POAF [45], our study highlighted the high variability and weak correlations between IL-6 levels and the occurrence of POAF. Mean IL-6 values were similar in patients with and without POAF in both surgical groups (150.38 vs. 186.84, 159 vs. 173.52). The elevated IL-6 levels likely reflect the overall degree of surgical stress [46]. They could also be influenced by factors like obesity, with which we found a correlation in our data (r = 0.7). This further supports the notion that while IL-6 robustly reflects systemic inflammation post-surgery, its specificity for predicting POAF, in isolation, might be limited within the context of the broader inflammatory response, particularly when compared to markers like NLR and CRP. While IL-6 is indeed a strong marker of systemic inflammation, serving as a key mediator in the acute-phase response to surgical trauma, its weak correlation with POAF suggests that systemic inflammatory markers, such as IL-6, may not directly predict the occurrence of POAF. The distinction lies in the fact that while IL-6 reflects the overall inflammatory milieu following surgery, it may not be sufficiently specific or sensitive to serve as a robust predictor of POAF in individual patients. Therefore, while IL-6 is a reliable indicator of systemic inflammation, its utility as a predictive marker for POAF is limited, and other factors, such as atrial tissue remodeling, electrical disturbances, or additional biomarkers, may contribute more significantly to predicting the risk of POAF.

Consistent with established risk factors, diabetes mellitus and blood transfusion were more prevalent in patients who developed POAF in both surgical groups. We observed a higher proportion of diabetic patients in the POAF groups (CABG: 38%, valve: 53%) than in non-POAF groups (CABG: 27%, valve: 11%).

Furthermore, blood transfusion was significantly more frequent among patients who developed POAF (CABG: 75%, valve: 84%) compared to those who did not. These findings underscore the relevance of diabetes mellitus and blood transfusion as important clinical risk factors for POAF, potentially acting through mechanisms involving enhanced systemic inflammation and oxidative stress [47-50]. Numerous studies have reported an increased incidence of POAF among diabetic patients undergoing cardiac surgery [26-29,32]. In our cohort, we also observed notable correlations between diabetes and postoperative inflammatory markers, with correlation coefficients of r = 0.50 between diabetes and CRP levels and r = 0.42 with NLR values at 24 hours, further supporting the link between diabetes, inflammation, and potentially worse postoperative outcomes, including arrhythmias. The observed association between blood transfusion and POAF is also consistent with studies highlighting the immunomodulatory and microcirculatory effects of allogeneic transfusions, which may contribute to POAF development [51,52]. The potential role of pre-existing anemia, possibly masked by diuretic use, in patients requiring transfusion also warrants further exploration. We acknowledge that coefficients in the range of r = 0.29 to 0.33 indicate only weak associations, suggesting that while inflammation may contribute to the risk of POAF, it is likely only one component within a multifactorial pathophysiological process. Age, CPB duration, diabetes, and blood transfusions can collectively influence the pathogenesis of postoperative atrial fibrillation through a multifactorial process involving age-related atrial remodeling, prolonged inflammatory responses elicited by CPB, impaired glucose metabolism, and heightened systemic inflammation associated with diabetes, and the pro-inflammatory effects and electrolyte disturbances induced by blood transfusions. These factors, individually and in combination, can contribute to atrial electrical remodeling, structural changes, and increased atrial susceptibility to arrhythmogenesis, thereby enhancing the risk of POAF development in the post-surgical period.

In relation to blood transfusions, 75% of patients in Group 1 and 84% in Group 2 who received transfusions developed postoperative POAF. The causes of POAF are unknown, but it is possible that patients who required transfusions had some degree of mild anemia before surgery that affected the occurrence of arrhythmias. Anemia is often underdiagnosed in the clinical setting, largely due to the diuretic treatment given to many patients. Diuretics, commonly used in clinical practice to treat conditions such as heart failure, hypertension, and edema, can cause a reduction in circulating blood volume. This may temporarily improve the clinical appearance of anemia by reducing plasma volume and thereby increasing hemoglobin and hematocrit levels. However, this effect does not address the underlying etiology of anemia and may mask the true severity of the condition. In patients with mild anemia, diuretics could potentially mask the hematologic abnormalities by artificially correcting the hemoglobin concentration through hemodilution, giving a false impression of adequate oxygen-carrying capacity. This phenomenon is particularly relevant in settings where clinical management of anemia is critical, such as the perioperative period or in patients with cardiovascular disease, where anemia may exacerbate adverse outcomes, including reduced tissue perfusion and increased risk of complications. Further research is needed to clarify how diuretics may affect the diagnosis and management of anemia, particularly in patients with concomitant cardiovascular or renal disease. Clinicians should be aware of this masking effect and consider using alternative diagnostic approaches, such as more accurate measurements of red blood cell mass or reticulocyte count when evaluating patients with suspected anemia. In addition, a comprehensive approach to anemia management should not only consider the hemodynamic effects of diuretic therapy but also aim to identify and address the underlying etiology, be it iron deficiency, chronic disease, or other hematologic disorders. The relationship between blood transfusion and the development of POAF following cardiac surgery remains incompletely understood. Nevertheless, numerous studies have demonstrated that the administration of allogeneic packed red blood cell (pRBC) transfusions, often used to correct anemia and compensate for intraoperative fluid loss, carries significant risks [51]. These risks are not confined to cardiac surgery; evidence from various surgical fields also supports an association between pRBC transfusions and increased incidence of POAF [53]. Allogeneic pRBC transfusions can lead to immunosuppression and microcirculatory issues. Some of the specific complications observed in cardiac surgery patients following transfusion include atrial fibrillation, infections (such as nosocomial pneumonia and sternal wound infections), myocardial infarction, and acute renal failure [8,11]. Regarding first-line treatment, all guidelines recommend a beta-blocker for the prevention of POAF. Other effective therapies for manifest AF included amiodarone and maintaining a plasma K+ concentration above 4.5 mmol/L. Cardioversion was indicated only when associated with hemodynamic instability. In contrast to preclinical studies [22,54-56] suggesting a role for IL-17A in POAF pathogenesis, our study did not find significant correlations between postoperative IL-17A levels and POAF occurrence. However, a significant correlation was identified between preoperative IL-17A levels and POAF. This could be due to several reasons, including the relatively small sample size of our study, the timing of IL-17A measurements, or potentially, IL-17A's role being more nuanced or relevant in specific subgroups of patients or at different stages of POAF development. Larger studies are needed further to elucidate the role of IL-17A in clinical POAF. Given the complexity of cardiac surgery involving CPB, the duration and administration of cardioplegia, ischemia-reperfusion syndrome, and calcium dysregulation, varying degrees of myocardial dysfunction are frequently observed [57]. An important question remains whether postoperative atrial fibrillation following cardiac surgery arises as a direct consequence of diastolic dysfunction (DD) or results from a complex interplay between DD and inflammation-induced cardiac remodeling, particularly affecting the left atrium or ventricle [36].

Diastolic dysfunction is a myocardial relaxation impairment characterized by elevated cardiac filling pressures, which adversely affect stroke volume [58]. The mechanisms through which diastolic dysfunction after cardiac surgery contributes to the development of AF are well-described and involve several interrelated physiological changes: increased atrial wall pressure, atrial afterload, atrial stretch [59], contractile dysfunction, and negative inotropic effects [7]. Although there is not yet a clear definition of low cardiac output syndrome after cardiac surgery, the most commonly cited criteria include a cardiac index (CI) < 2.0 L/min/m^2^, the use of inotropic agents for more than 30 minutes to maintain systolic blood pressure (SBP) > 90 mm Hg, CI < 2.2 L/min/m^2^ combined with lactate > 2.0 mmol/L, and the requirement for intra-aortic balloon pump (IABP) or mechanical circulatory support [57,60]. In our study cohort (Group 1), we observed a statistically significant negative correlation between CPB time and postoperative EF (r = 0.49, *P* ≤ 0.05), suggesting that prolonged CPB duration may adversely impact EF and contribute to increased postoperative morbidity and mortality following cardiac surgery [61]. Diastolic dysfunction and postoperative atrial fibrillation further compromise cardiac output, increasing the likelihood of acute heart failure [62,63] with specific implications for patient outcomes: perioperative morbidity [64], hospital length of stay, acute renal failure [65], respiratory failure with prolonged hours of mechanical ventilation, thrombotic risk [66]. Patients who develop POAF after cardiac surgery have a 33% higher risk of HF-related hospitalization over a 1.7-year follow-up period [64] or even at 5 years [63]. Future studies are needed to observe whether DD causes acute heart failure with increased inflammatory markers implicating POAF due to low cardiac output. In our study, we identified statistically significant correlations between the inflammatory markers examined and ejection fraction. The change in CRP, measured preoperatively, at 24 and 48 hours postoperatively, demonstrated a correlation with EF of r = 0.7 (*P* ≤ 0.05). The correlation between CRP levels and EF suggests that elevated CRP levels may be associated with impaired heart function, which aligns with the understanding that inflammation plays a crucial role in cardiovascular dysfunction, particularly in conditions such as heart failure. However, it is essential to recognize that CRP is not heart-specific and can be elevated due to various other conditions. Therefore, while CRP may correlate with EF, its value as a direct indicator of myocardial dysfunction is limited. Moreover, CRP levels are influenced by a range of factors, making it difficult to establish a definitive causal link to EF without considering additional variables.

Additionally, the level of IL-17A at 48 hours postoperatively was correlated with EF, showing a correlation coefficient of r = 0.63 (*P* ≤ 0.05). This suggests that postoperative inflammation may predispose to POAF, leading to acute heart failure and low cardiac output. IL-17A is a cytokine involved in inflammation, with a known role in cardiovascular diseases through its impact on vascular inflammation and myocardial remodeling. A negative correlation between IL-17A levels and EF could indicate that higher IL-17A is linked to lower EF, possibly reflecting inflammation-induced myocardial dysfunction. However, it is essential to note that correlation does not imply causation, and other factors, such as comorbid conditions or pre-existing heart disease, may also influence both IL-17A levels and EF. More in-depth longitudinal or experimental research is necessary to determine whether IL-17A directly contributes to reduced EF or merely serves as a marker of systemic inflammation associated with heart failure. Adequate studies on larger groups of patients are necessary to determine whether these inflammatory markers can be used as predictors in POAF-induced acute heart failure after cardiac surgery. Given the increased inflammatory response following cardiac surgery, the new onset of heart failure, and the associated pathologies of the patients, it is difficult to differentiate which is the trigger responsible for the increase in inflammatory markers levels.

Our study has several limitations, including its single-center, observational design, and moderate sample size, which necessitate validation in larger, multi-center cohorts. The focus on elective cardiac surgery limits generalizability to higher-risk and emergency settings. This study is restricted to the perioperative period, with a particular focus on the initial days of intensive care, and does not include long-term patient follow-up. We assessed a limited panel of inflammatory markers; a more comprehensive assessment of the inflammatory and immune response, including other cytokines, chemokines, and immune cell subsets, might provide a more complete picture. Finally, while we found associations, this study cannot establish causality.

Despite the acknowledged limitations, our study possesses several strengths that enhance the robustness and clinical relevance of our findings. Firstly, the prospective study design enabled systematic and standardized data collection, thereby minimizing recall bias and ensuring a consistent approach to POAF detection and biomarker measurement. Secondly, by focusing on patients undergoing elective cardiac surgery with hemodynamic stability during CPB, we investigated a clinically relevant and well-defined patient population, enhancing the homogeneity of our cohort and the applicability of our findings to this common surgical setting. Thirdly, the assessment of a focused panel of inflammatory markers (NLR, CRP, IL-6, and IL-17A) allowed us to evaluate a range of inflammatory pathways, from readily accessible systemic markers like NLR and CRP to more specific cytokines, providing a multi-faceted perspective on the inflammatory milieu in POAF. Furthermore, the inclusion of both preoperative and postoperative biomarker measurements, particularly the investigation of preoperative IL-17A, provides valuable insights into the temporal dynamics of inflammatory responses and potential baseline risk factors. Although systematic monitoring for POAF was performed for seven days postoperatively, we acknowledge that atrial fibrillation can occasionally develop later, particularly among patients undergoing valve surgery, which may have led to an underestimation of POAF incidence. While our strict exclusion criteria enhanced internal validity by reducing confounding, they limit the generalizability of findings to broader populations, such as elderly patients with multiple comorbidities or those undergoing urgent surgery. Additionally, while elevated preoperative NLR values were observed in some patients, we retained these subjects in the analysis to maintain sample size and statistical power. Future larger studies could explore excluding patients with markedly elevated baseline inflammation to assess potential improvements in biomarker predictive accuracy. Furthermore, given that EF was significantly lower in POAF patients and correlated with inflammatory markers such as CRP and IL-17A, EF may act as a confounder in the observed associations between inflammation and POAF. Although multivariate adjustment for EF was limited by sample size constraints in this study, future larger-scale investigations should account for this potential confounding effect. Finally, the focus on readily accessible and clinically practical biomarkers, such as NLR and CRP, also enhances the translational potential of our study for future risk stratification strategies in routine cardiac surgery practice.

Our findings suggest that postoperative CRP at 48 hours and NLR at 24 hours warrants further investigation as a readily accessible marker to potentially identify patients at increased risk of POAF following cardiac surgery. Specifically, in the coronary cohort, NLR at 24 and 48 hours and CRP at 48 hours demonstrated modest predictive performance (AUC up to 0.69 for NLR). While individual inflammatory markers, such as IL-6, may not be sufficient predictors on their own, CRP and NLR, which reflect a broader systemic immune balance, may offer a more integrated and clinically useful assessment, especially in CABG patients.

## CONCLUSION

Our study provides preliminary evidence that postoperative CRP levels at 48 hours and NLR at 24 hours could serve as potential markers for identifying patients at increased risk of postoperative atrial fibrillation following cardiac surgery. These markers, particularly NLR in the coronary cohort, demonstrated modest discriminatory ability, with an AUC of up to 0.69. However, this suggests that while NLR and CRP may be useful tools, their sensitivity and specificity remain limited, necessitating further validation in larger, multi-center cohorts. Notably, individual markers, such as IL-6 and IL-17A, did not demonstrate strong independent predictive value for POAF in our study.
